# Ferroptosis and Its Role in Epilepsy

**DOI:** 10.3389/fncel.2021.696889

**Published:** 2021-07-15

**Authors:** Yuxiang Cai, Zhiquan Yang

**Affiliations:** Department of Neurosurgery, Xiangya Hospital, Central South University, Changsha, China

**Keywords:** ferroptosis, epilepsy, iron, lipid peroxidation, GSH, GPx

## Abstract

Epilepsy is one of the most common symptoms of many neurological disorders. The typical excessive, synchronous and aberrant firing of neurons originating from different cerebral areas cause spontaneous recurrent epileptic seizures. Prolonged epilepsy can lead to neuronal damage and cell death. The mechanisms underlying epileptic pathogenesis and neuronal death remain unclear. Ferroptosis is a newly defined form of regulated cell death that is characterized by the overload of intracellular iron ions, leading to the accumulation of lethal lipid-based reactive oxygen species (ROS). To date, studies have mainly focused on its role in tumors and various neurological disorders, including epilepsy. Current research shows that inhibition of ferroptosis is likely to be an effective therapeutic approach for epilepsy. In this review, we outline the pathogenesis of ferroptosis, regulatory mechanisms of ferroptosis, related regulatory molecules, and their effects on epilepsy, providing a new direction for discovering new therapeutic targets in epilepsy.

## Introduction

Epilepsy is one of the most common brain conditions and is characterized by spontaneous recurrent and transient central nervous system (CNS) dysfunction resulting from excessive synchronous discharge of brain neurons. Epilepsy has high morbidity and mortality rates and affects over 70 million people worldwide; nearly 80% live in low- and middle-income countries (Saxena and Li, 2017; Thijs et al., 2019). Epileptic seizures have numerous neurobiological, cognitive, and psychosocial consequences such as severely affecting the employability and social communication level of patients with epilepsy, as well as reducing their quality of life (Fisher et al., 2014; Leeman-Markowski and Schachter, 2016). Pathophysiologically, seizures primarily result from abnormal activity in cortical neurons; however, glial cells and myelinated axons may also be involved (Fisher, 1995). This is a self-facilitated pathological process that ultimately leads to the loss of excitatory and inhibitory neurons in specific subfields, axonal sprouting and synaptic reorganization, and alterations in glial function and structure (Borges et al., 2003; Gan et al., 2015; Thijs et al., 2019; Vivash et al., 2011). “Epilepsy” is an umbrella term for a variety of disorders that occur as a result of brain dysfunction that may result from many different causes (Fisher et al., 2005). Therefore, many different neurobiological processes have been implicated as potential treatment targets for epileptogenesis (Kobow et al., 2012). Antiepileptic drugs (AEDs) are the main treatment modalities for epilepsy. However, up to one-third of patients with epilepsy have drug-refractory epilepsy (Golyala and Kwan, 2017). Moreover, at present, the mechanism of epilepsy is not yet clear, which poses a great challenge to the treatment of epilepsy.

Cell death can be classified as accidental or regulated cell death. Accidental cell death occurs in response to severe physical, chemical and mechanical insults and cannot be reversed by molecular signaling pathways (Stockwell et al., 2017). In contrast, the regulation of cell death is mediated by different molecular signaling pathways. Various forms of regulated cell death have been defined, including apoptosis, necroptosis, pyroptosis, autophagy, and ferroptosis. These forms all play an essential role in maintaining homeostasis when an organism suffers from disturbances in the intracellular or extracellular microenvironment (Tang et al., 2019). Ferroptosis is a newly defined form of regulated cell death and was first described by Dixon et al. (2012). Specifically, it is characterized by the accumulation of intracellular iron ions, leading to the accumulation of lethal lipid-based reactive oxygen species (ROS). Ferroptosis has been extensively reported to be involved in various neurological disorders, including traumatic brain injury, stroke, Alzheimer’s disease, Parkinson’s disease, Huntington’s disease, and brain tumors (Do Van et al., 2016; Chen et al., 2017; Alim et al., 2019; Xie et al., 2019; Ashraf et al., 2020; Kumar et al., 2020). Ferroptosis has also been detected in epilepsy (Kahn-Kirby et al., 2019). However, its specific role and mechanism in epilepsy remain unclear. Understanding the regulatory mechanisms of ferroptosis in epilepsy will provide a new approach to prevent and treat this disease. In this article, we review the mechanism of ferroptosis and its role in epilepsy and provide a new direction for discovering new therapeutic targets.

## Ferroptosis

**FIGURE 1:**
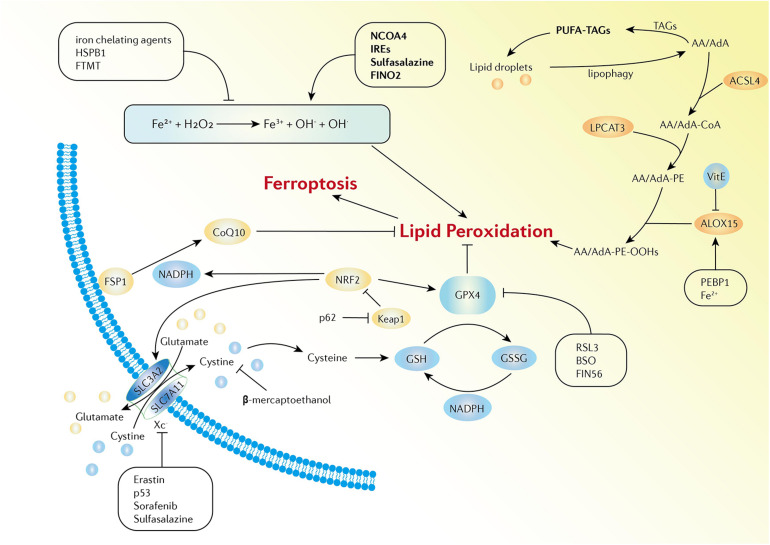
Activation and inhibition mechanisms of ferroptosis. The Fe-mediated Fenton reaction, peroxidation of PUFA, and the redox of GSH/GPX4/system Xc- are the main biochemical events involved in ferroptosis. Excessive Fe^2+^ can donate electrons to generate hydroxyl radicals, which have high reactivity with biological molecules, leading to lipid peroxidation and the eventual development of ferroptosis. Activators and inhibitors in each stage of cellular iron metabolism, including iron uptake (e.g., IREs, HSPB1, and sulfasalazine), export (e.g., IREs), storage (e.g., FTMT and NCOA4), and turnover (e.g., iron chelating agents and FINO2), have close relations with ferroptosis. PUFAs are esterified into membrane phospholipids which then react with ROS and finally facilitate ferroptosis in the cells. Iron can increase the activity of ALOXs. Vitamin E could compete with ALOX at the substrate binding site against ferroptosis. PEBP1 increases the catalytic activity of ALOX15 by combining itself with ALOX15. GPX4, GSH, and system X_c_^–^ are the main regulators of ferroptosis. System Xc- can be regulated at transcriptional and post-transcriptional stages by various ferroptosis inducers. p53 inhibits SLC7A11 expression. Erastin, sorafenib, and sulfasalazine can bind and inactivate SLC7A11. β-mercaptoethanol inhibits erastin-induced ferroptosis by increasing the intracellular concentration of cystine. BSO is an inhibitor of GSH biosynthesis, FIN56 promotes the degradation of GPX4, and RSL3 binds to GPX4 to directly inactivate GPX4. In addition, the FSP1-CoQ10-NADPH pathway is a stand-alone parallel anti-ferroptotic pathway. The NRF2-Keap1 protein complex also plays an important role in mediating lipid peroxidation and ferroptosis.

Ferroptosis is defined as iron-dependent programmed cell death that is different from traditional cell death processes, such as apoptosis and autophagy. The morphological changes of ferroptosis do not conclude loss of plasma membrane integrity, swelling of cytoplasmic organelles, or chromatin condensation. Increased mitochondrial membrane density and reduction of mitochondrial membrane density are typical morphological changes (Bayır et al., 2020). Ferroptosis is characterized by the iron-dependent accumulation of free radicals and lipid oxidation products and is regulated by various cell signaling pathways and genes. Although the specific regulatory network is not clear, three main factors are involved in ferroptosis: abnormal metabolism of iron ions, depletion of the redox glutathione (GSH)/glutathione peroxidase 4 (GPX4)/system X_c_^–^, and aberrant lipid peroxidation (Dixon et al., 2012; Yang and Stockwell, 2016; Stockwell and Jiang, 2020; Figure 1).

## Iron Metabolism and Ferroptosis

**FIGURE 2:**
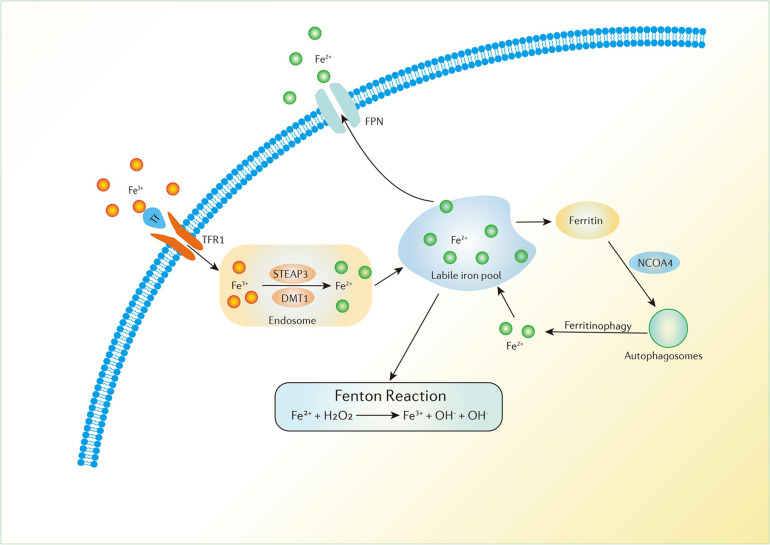
Iron metabolism in the human body. Fe^3+^ can be delivered into the cells by the binding of Tf and TFR1 in the cell membrane which is then transported to the endosome. Fe^3+^ is converted to Fe^2+^ in the endosome and is then released to a labile iron pool. Iron can be stored in ferritin in the cytoplasm and can also be exported by FPN. Ferritinophagy can modulate sensitivity to ferroptosis by the degradation of ferritin. Excess Fe^2+^ can donate electrons to lipid peroxidation via the Fenton reaction.

Iron is a necessary element in cellular metabolism, energy generation, and growth in organisms. It is found in the human body in both ferrous (Fe^2+^) and ferric (Fe^3+^) forms. The conversion between the two forms gives iron the capability to accept and donate electrons. Because of its ability to donate electrons, proteins containing Fe^2+^ always serve as cofactors and catalysts in various oxidation-reduction reactions. In addition, iron tends to be stored and transported in the Fe^3+^ form (Lei et al., 2019). In the blood, Fe^3+^ binds to transferrin (Tf) to form a complex which can be delivered into the cells by binding to transferrin receptor-1 (TFR1) in the cell membrane which is then transported to the endosome (Andrews and Schmidt, 2007). In the endosome, Fe^3+^ is converted to Fe^2+^ by oxidation-reduction and is then released into a labile iron pool in the cytoplasm. The six-transmembrane epithelial antigen of prostate 3 (STEAP3) and divalent metal transporter 1 (DMT1) are involved in this process (Ohgami et al., 2005). The labile iron pool is present in mitochondria, lysosomes, cytosol and the nucleus and can be regulated by the absorption, distribution and export of iron in the cell (Galaris et al., 2019). Biochemically, iron can be stored in ferritin in the cytoplasm, which includes ferritin light chain (FTL) and ferritin heavy chain 1 (FTH1). Iron can also be exported by ferroportin (FPN), an iron efflux pump in the cellular membrane, which can oxidize Fe^2+^ to Fe^3+^ (Cao and Dixon, 2016). All the processes described above maintain iron homeostasis in the body (Figure 2).

Excess iron in cells promotes ferroptosis. Recent studies have indicated that excess Fe^2+^ can donate electrons to hydrogen peroxide (H_2_O_2_) to generate hydroxyl radicals, which have high reactivity with biological molecules, such as proteins, lipids, and nucleic acids, leading to lipid peroxidation and the eventual development of ferroptosis (Stockwell et al., 2017). Moreover, Fe^2+^ is also involved in the catalytic subunit of lipoxygenase (ALOX). Iron-dependent ALOX enzymes can generate ROS to catalyze the oxidation of polyunsaturated fatty acids (PUFAs) (Dixon et al., 2015). Ferroptosis requires iron from the extracellular environment. Therefore, each stage of cellular iron metabolism, including iron uptake, export, storage, and turnover, is closely related to ferroptosis. Iron regulatory proteins 1 and 2 (IRP1 and IRP2) and iron-responsive elements (IREs) are master transcription factors of DMT1, TFR1, FTH1, FTL, and FPN, all of which are involved in maintaining cellular iron homeostasis (Anderson et al., 2012). Suppression of IREs increases the gene expression of FTH1 and FTL, which can limit erastin-induced ferroptosis (Dixon et al., 2012). Overexpression of TF and TFR1 enhances iron uptake, which makes cells sensitive to ferroptosis (Anderson et al., 2012). Exogenous sources of iron enhance erastin-mediated ferroptosis such as ferric ammonium citrate, ferric citrate, and iron chloride hexahydrate. However, iron chelating agents, such as deferoxamine, desferrioxamine mesylate, and ciclopirox, can combine with free iron ions to form stable compounds, thereby blocking lipid ROS and inhibiting the process of ferroptosis (Dixon et al., 2012). Autophagy can modulate the sensitivity to ferroptosis via the selective autophagy of ferritin; this process is called “ferritinophagy.” Nuclear receptor coactivator 4 (NCOA4) plays an essential role in this process. Specifically, NCOA4 can selectively bind to ferritin and then deliver it to autophagosomes for lysosomal degradation. Fe^2+^ is released in the cell by degradation, which promotes ferroptosis (Hou et al., 2016). In addition, heat shock protein beta-1 (HSPB1), a type of small heat shock protein, is found to be a negative regulator of ferroptotic cancer cell death. Overexpression of HSPB1 induced by protein kinase C inhibits TFR1-mediated iron uptake, which reduces the level of intracellular iron. However, HSPB1 knockdown increases erastin-induced iron uptake in cancer cells (Sun et al., 2015). Mitochondrial ferritin (FTMT) is an iron-storage protein that is located in the mitochondria. Overexpression of FTMT directly incorporates iron in the mitochondria to inhibit erastin-induced ferroptosis (Wang et al., 2016). Taking all research into account, iron metabolism serves as an essential mechanism of ferroptosis regulation.

## Lipid Peroxidation and Ferroptosis

Numerous lipid species are distributed in intra- or extra-cellular area and play important roles in the energy supply and structural components of the intracellular membrane system. Moreover, lipid metabolism serves as a key mediator of many signaling processes, including the regulation of ferroptosis. PUFAs contain bis-allylic hydrogen atoms that can be readily abstracted. Therefore, PUFAs are good substrates for lipid peroxidation. PUFAs are esterified into membrane phospholipids, react with ROS and facilitate ferroptosis in cells. The accumulation of lipid peroxidation is promoted by abundant PUFAs in cells, especially under oxidative stress conditions (Yang et al., 2016). Among thousands of molecular species of PUFAs, arachidonic acid (AA) and its elongation product, adrenic acid (AdA), are the main substrates of lipid peroxidation in ferroptosis (Kagan et al., 2017). Three enzymes are involved in the process of lipid peroxidation: acyl-CoA synthetase long-chain family 4 (ACSL4), lysophosphatidylcholine acyltransferase 3 (LPCAT3), and lipoxygenases (ALOXs). First, ACSL4 binds to AA/AdA and catalyzes the esterification reaction to produce AA/AdA-CoA derivatives. LPCAT3 catalyzes the biosynthesis of AA/AdA-CoA and membrane phospholipids, such as phosphatidylethanolamine (PE), to form AA/AdA-PE (Kagan et al., 2017; Shintoku et al., 2017). Enzymatic oxidation of AA/AdA-CoA is induced by ALOXs, which are enzymatic effectors that contain iron in their catalytic region. There are six different isoforms of the mammalian ALOX family: ALOXE3, ALOX5, ALOX12, ALOX12B, ALOX15, and ALOX15B. Among them, ALOX15 can selectively and specifically induce the oxidation of AA/AdA-PE, resulting in the enzymatic production of AA/AdA-PE-OOHs (Hinman et al., 2018). The catalytic activity of ALOX15 depends on the formation of a complex of ALOX15 with a scaffold protein, phosphatidylethanolamine-binding protein 1 (PEBP1) (Wenzel et al., 2017). The cleavage of AA/AdA-PE-OOHs leads to the accumulation of highly electrophilic secondary oxidation products, such as epoxy, oxo- or aldehyde groups, which could cause the formation of pores in the lipid bilayer, and rupture of the plasma membrane (Reichert et al., 2020).

In brief, lipid peroxidation forms toxic lipid free radicals and serves as a trigger for ferroptosis, especially when excess iron ions exist in the cytoplasm. The higher concentration of PUFAs in the cells causes a higher degree of lipid peroxidation, leading to the aggravation of ferroptosis. Conversely, ferroptosis can be blocked by modulating enzymes involved in the biosynthesis of lipid peroxidation (Doll et al., 2017). In addition to AA and AdA, other long-chain PUFAs can also induce ferroptosis when the highly electrophilic secondary oxidation products produced by them reach the threshold (Shah et al., 2018). Triacylglycerols (TAGs) are traditionally known as energy storage molecules. However, TAGs can also store excess PUFAs by forming PUFA-TAG compounds, which are specifically stored in lipid droplets in cells. This process prevents the formation of lipid peroxides and subsequent ferroptosis by separating PUFAs from cellular membranes. In contrast, the autophagic degradation of intracellular lipid droplets, i.e., “lipophagy,” increases the production of PUFAs and promotes ferroptotic cell death (Bai et al., 2019). The expression of ACSL4 in ferroptosis-resistant cells has been found to be remarkably decreased compared to that in ferroptosis-sensitive cells. However, overexpression of ACSL4 via gene transfection restored sensitivity to erastin-induced ferroptosis in ferroptosis-resistant cells (Yuan et al., 2016). Additionally, the inhibition of GPX4 was thought to accelerate uncontrolled lipid peroxidation, leading to cell death, whereas GPX4 and ACSL4 double knockout cells had significantly prolonged overall survival (Doll et al., 2017). These results indicate that ACSL4 is not only a marker of ferroptosis sensitivity, but also that it plays an important functional role in ferroptosis. Various forms of vitamin E have the ability to scavenge hydroxyl group radicals, which can also protect cells against ferroptotic death by competing with ALOX at the substrate-binding site (Kagan et al., 2017). Silencing the gene expression of ALOXs depletes the substrates required for lipid peroxidation and increases resistance to ferroptosis under GSH depletion conditions. Intriguingly, this lethal effect of ALOXs is negligible when the intracellular GSH level is normal (Yang et al., 2016). Accordingly, among the common ferroptosis-inducing agents, only parts of them are inhibited by ALOX depletion.

## GPX4 and Ferroptosis

GPXs are traditionally known as enzymes that catalyze the reduction of inorganic or organic hydrogen peroxide. There are eight members (GPX1–GPX8) in mammalian GPX family (Brigelius-Flohé and Maiorino, 2013). GPX4 can not only reduce inorganic hydrogen peroxide, but also neutralize phospholipid, cholesterol and cholesterolester hydroperoxides, even when anchored in cellular membranes (Brigelius-Flohé and Maiorino, 2013). Therefore, GPX4 and its substrate GSH are regarded as the main regulators of ferroptosis. GPX4 is a selenoprotein that contains selenocysteine in its catalytic center. The catalytic center of selenocysteine has three different redox states depending on the intracellular conditions, which affect the activity of GPX4 (Ingold et al., 2018). Lipid peroxides are reduced to non-toxic lipid alcohols by the spontaneous oxidation of selenium in GPX4. As a necessary substrate in the process, reduced GSH is converted to oxidized glutathione (GSSG), accompanied by the formation of active GPX4 (Cozza et al., 2017). GSH is a small molecule that is formed by glutamic acid, cysteine, and glycine. With a wild distribution, GSH is closely involved in the cellular antioxidant stress system. Additionally, GSH can prevent iron oxidation by directly binding to Fe^2+^ in the labile iron pool (Hider and Kong, 2011). The biosynthetic efficiency of GSH is closely related to the intracellular concentration of cysteine. Normally, cysteine is transported into cells in its oxidized form (cystine) (Kagan et al., 2017). This import process is executed by system X_c_^–^, a Na^+^-independent cystine/glutamate reverse transporter on the cell membrane (Dixon et al., 2012). System X_c_^–^ belongs to the heteromeric amino acid transporter family, which consists of two components linked by disulfide: the light-chain subunit solute carrier family 7 member 11 (SLC7A11) and the heavy-chain subunit solute carrier family 3 member 2 (SLC3A2) (Sato et al., 1999). Cysteine is imported into the cells and glutamate is exported at the same time through system X_c_^–^. Consequently, the GPX4-GSH-cysteine redox axis is a key regulator of lipid peroxidation and ferroptosis. The decreased absorption of cystine inhibits the synthesis of GSH, and the deficiency of GSH inactivates GPX4, which leads to a diminished reduction ability, resulting in the accumulation of lipid peroxides and ferroptosis. System X_c_^–^ can be regulated at the transcriptional and post-transcriptional stages by various ferroptosis inducers, such as p53, erastin, sorafenib, sulfasalazine, and glutamate (Tobaben et al., 2011; Dixon et al., 2012, 2014; Dahlmanns et al., 2017; Yu et al., 2019). Among these molecules and drugs, p53 can inhibit SLC7A11 expression (Dixon et al., 2014). Moreover, erastin, sorafenib, and sulfasalazine can bind and inactivate SLC7A11 (Dixon et al., 2012; Dahlmanns et al., 2017; Yu et al., 2019). In addition, sulfasalazine can aggravate iron accumulation by enhancing TFR1 expression (Yu et al., 2019). In contrast, β-mercaptoethanol inhibits erastin-induced ferroptosis by increasing the intracellular concentration of cystine (Takahashi et al., 2002). Buthionine sulfoximine (BSO) is an inhibitor of GSH biosynthesis and directly affects the activity of GPX4, leading to oxidative stress and cell death (Slott and Hales, 1987). GPX4 inhibitors are also important regulators of ferroptosis. Ferroptosis inducing 56 (FIN56) induces ferroptosis by promoting the degradation of GPX4 and suppressing coenzyme Q_10_ (CoQ_10_) (Shimada et al., 2016). The concentration of GPX4 was found to be reduced when FINO_2_ was applied to cells. However, unlike other ferroptosis inducers, FINO_2_ does not directly inhibit or deplete GPX4. Instead, FINO_2_ directly oxidizes Fe^2+^ and lipids to induce lipid peroxidation (Gaschler et al., 2018). RAS-selective lethal small molecule 3 (RSL3) binds to the nucleophile moiety of selenocysteine at the active site of GPX4, which directly inactivates GPX4 and triggers ferroptosis (Sui et al., 2018).

## Other Pathways and Processes of Ferroptosis

Apoptosis-inducing factor mitochondria-associated 2 (AIFM2) is known to induce apoptosis in mitochondria. AIFM2 has been renamed “ferroptosis suppressor protein 1” (FSP1) in recent studies because FSP1 was found to inhibit ferroptosis when intracellular GPX4 is deficient (Bersuker et al., 2019; Doll et al., 2019). FSP1 catalyzes the regeneration of CoQ_10_ with nicotinamide adenine dinucleotide phosphate (NADPH). NADPH is an essential reducing agent that regulates lipid hydroperoxide levels, while CoQ_10_ is involved in electron transport in the mitochondrial respiratory chain. Moreover, as an endogenous lipophilic antioxidant, CoQ_10_ also inhibits lipid peroxidation by neutralizing free radical intermediates (Bersuker et al., 2019). In addition, when GSSG is recycled into GSH, NADPH serves as a cofactor in this reduction reaction (Xiao et al., 2018). Therefore, the FSP1-CoQ_10_-NADPH pathway is a stand-alone parallel anti-ferroptotic pathway that cooperates with GPX4 and GSH (Doll et al., 2019).

Nuclear factor erythroid 2-related factor 2 (NRF2) is a transcription factor that regulates the expression of related genes. Kelch-like ECH-associated protein 1 (Keap1) is an endogenous inhibitor of NRF2 and can sequester NRF2 and mediate proteasomal degradation under basal conditions (Bellezza et al., 2018). Under oxidative stress condition, NRF2 detaches from Keap1 and translocates into the nucleus to modulate the cellular antioxidant response by increasing the expression of target genes involved in the metabolism of ROS, such as GPX4 and SLC7A11 (Qiang et al., 2020). In addition, the activation of NRF2 can also upregulate cellular NADPH, promote iron storage, and reduce cellular iron uptake (Kovac et al., 2015; Han et al., 2021). Therefore, the NRF2-Keap1 protein complex plays an important role in mediating lipid peroxidation and ferroptosis. The expression of p62 competitively binds to Keap1 to prevent NRF2 degradation, which promotes cell resistance to ferroptosis (Sun et al., 2016).

## Ferroptosis and Epilepsy

Epilepsy has a major burden on many patients in terms of quality of life and risk of premature mortality. In addition, the burden on family is not negligible. However, because the basic pathophysiology of epilepsy is not yet fully understood, a more in-depth study is urgently needed. Previous studies have mainly focused on the role of ferroptosis in neoplastic and some neurodegenerative diseases, such as Alzheimer’s disease, Parkinson’s disease, and Huntington’s disease (Do Van et al., 2016; Ashraf et al., 2020; Kumar et al., 2020). Recently, researchers have focused on the significant effects of ferroptosis on the pathophysiology of epilepsy.

## Links Between Ferroptosis and Epilepsy

Epilepsy is characterized by excessive, synchronous and aberrant firing of neurons originating from different cerebral cortices; in general, there is no obvious predisposing cause (Thijs et al., 2019). The abnormal firing of neurons in epileptic patients, especially in those who continue to experience seizures, leads to various pathological changes at the cellular level. Excessive oxidative stress is one of the most typical pathological changes associated with epileptic seizures (Engelborghs et al., 2000). Interestingly, of the organs, the brain is most vulnerable to oxidative stress because of its high consumption of oxygen. In addition, the brain contains many PUFAs in the neuronal membrane, which are targets of lipid peroxidation (Perry et al., 2002). Iron is also abundant in the brain and is involved in the formation of hydroxyl radicals (Valko et al., 2007). Therefore, overproduction of oxidative stress in the brain leads to lipid peroxidation, epileptic activity, and neuronal death (Lin et al., 2020). Due to the essential role of oxidative stress and lipid peroxidation in inducing ferroptosis, these epileptic pathological processes in the brain are closely related to ferroptosis. Iron overload is a common cause of hemorrhagic post-stroke epilepsy and post-traumatic epilepsy (Mori et al., 1990; Ikeda, 2001). Injection of hemoglobin or iron salts into the rat cortex is known to establish an animal model of chronic epileptic focus. After ferric chloride injection into the rat cerebral cortex, intracellular superoxide anion and hydroxyl radicals are increased. Subsequently, lipid peroxidation in neuronal membranes is activated, leading to chronic recurrent seizure (Mori et al., 1990). Moreover, transferrin saturation has been found to be significantly higher in patients with epilepsy than in controls, which enhances iron uptake and makes cells sensitive to ferroptosis (Ikeda, 2001; Anderson et al., 2012). Another study showed that lipid peroxidation in patients with epilepsy was significantly higher than that in controls. Furthermore, plasma vitamin A, E, and C concentrations were within the normal range in patients with epilepsy who were treated with phenobarbital who did not experience seizures for 1 year (Sudha et al., 2001). The improvement in antioxidant status suggests that lipid peroxidation may be involved in epilepsy. In addition, ALOX15 can induce a series of destructive events when cells are subjected to glutamate-induced oxidative stress, including breakdown of the mitochondrial membrane potential, production of ROS, and cytochrome c release, finally leading to cell death (Pallast et al., 2009). Uncontrolled recurrent seizures can increase ROS production in the heart and cause cardiomyocyte ferroptosis, acting as an underlying mechanism of sudden unexpected death in epilepsy (Akyuz et al., 2021). Collectively, activation of the ferroptosis pathway is implicated in the pathogenesis of epilepsy and epileptic neuronal death.

Ferroptosis is also associated with cognitive impairment in temporal lobe epilepsy (TLE); specifically, the accumulation of a large amount of lipid peroxides and the depletion of GSH have been detected in kainic acid-induced TLE in rats, accompanied by a reduction in the mitochondrial area of hippocampus neuron (Ye et al., 2019). Nonetheless, as the most important free radical scavenging compound and physiological regulator in ferroptosis, GPX4 and GSH may have key roles in attenuating oxidative stress and preventing neuronal death in epilepsy. Patients with epilepsy show low levels of GPX4 and GSH compared to healthy controls (Mueller et al., 2001). The persistently low levels of GPX4 and GSH lead to neuronal excitability changes, hippocampal neuron loss, and astrocyte proliferation (Kurzatkowski and Trombetta, 2013). Trimethyltin is a toxic organotin compound that can induce hippocampal CA3 damage and increase aggression, seizure susceptibility, and memory deficits in rats. These harmful effects of trimethyltin are implicated in the decreased expression levels of GSH and GPX4 (Shin et al., 2005). As a traditional anti-cancer drug, lapatinib can modulate oxidative stress and inhibit tumor progression. Recently, lapatinib was found to prevent kainic acid-induced seizures and ferroptosis in mice by restoring GPX4 (Jia et al., 2020).

Interestingly, ferroptosis pathway-related molecules are also affected by AEDs and other compounds. Epileptic children receiving therapeutic doses of levetiracetam showed significantly elevated levels of lipid peroxidation (Haznedar et al., 2019). Valproic acid (VPA) is a short-branched chain fatty acid that is associated with increased lipid hydroperoxides and decreased GSH levels. However, melatonin prevents VPA-induced GSH decrease and lipid hydroperoxides, reflecting the neuroprotective effect of melatonin (Chaudhary and Parvez, 2018). In epileptic patients receiving phenytoin monotherapy, the level of serum malondialdehyde was significantly increased, whereas the GSH level was significantly decreased. However, no significant changes in these parameters were observed in epileptic patients treated with carbamazepine or lamotrigine monotherapy, which were found to result in less disturbance to lipid peroxidation (Liu et al., 1998; Sarangi et al., 2016). Selenium and topiramate combination supplementation increases erythrocyte GSH and GPX4 and plasma concentrations of vitamins A and C in epileptic patients (Yürekli and Nazıroğlu, 2013). Oxcarbazepine monotherapy decreases lipid peroxidation levels in epileptic patients; this antioxidation may play a role in the mechanism underlying the antiepileptic effects of oxcarbazepine treatment (Arhan et al., 2011).

## Regulation of Ferroptosis in the Treatment of Epilepsy

Current research suggests that various proteins, drugs, and signaling pathways provide different levels of neuroprotection in epilepsy and alleviate the frequency of seizures by targeting ferroptosis pathway-related molecules. Deferoxamine (DFO) is an iron chelator that efficiently clears iron. DFO treatment in ferric chloride-induced epilepsy has been found to decrease local transferrin and significantly suppress epilepsy (Zou et al., 2017). Similarly, ferrostain-1, a specific inhibitor of ferroptosis, was found to attenuate cognitive impairment in epileptic rats by inhibiting P38 mitogen-activated protein kinase activation (Ye et al., 2020). Therefore, targeting abnormal iron metabolism may be an effective treatment for inhibiting the occurrence and development of epilepsy. Polyphenols are naturally occurring ROS scavenging compounds. Recently, curcumin and epigallocatechin-3-gallate (EGCG) have been regarded as novel ferroptosis inhibitors, which might protect cells against ferroptosis by acting as iron chelators and preventing GSH depletion, GPX4 inactivation, and lipid peroxidation (Kose et al., 2019). Inhibition of ferroptosis may be induced by activating the NRF2-Keap1 pathway (Lin et al., 2019). In addition, RTA 408 activates NRF2 by inhibiting Keap1, leading to the inhibition of lipid peroxidation, mitochondrial depolarization, and ferroptosis in an *in vitro* model of seizure-like activity (Shekh-Ahmad et al., 2019). The activation of NRF2 prevents the development of spontaneous seizures and alleviates the severity of epilepsy (Shekh-Ahmad et al., 2018). The flavonoid compound apigenin can relieve myeloperoxidase-mediated oxidative stress and inhibit ferroptosis of neuronal cells in epileptic mice (Shao et al., 2020). N-acetylcysteine (NAC) is an acetylated precursor of GSH. The GSH level is increased and hippocampal neuron loss is decreased when NAC and sulforaphane combination treatment is applied to epileptic rats. Consequently, the onset of epilepsy was significantly delayed, and disease progression was partly blocked (Pauletti et al., 2019). The clinical-stage therapeutic vatiquinone (EPI-743, α-tocotrienol quinone) has been reported to reduce seizure frequency and associated morbidity in mitochondrial disorders. Further research indicates that EPI-743 reduces seizure incidence by preventing ferroptosis in GSH depletion and iron overload condition (Kahn-Kirby et al., 2019). CoQ10 is an antioxidant compound that ameliorates spontaneous recurrent seizures and inhibits hippocampal neuronal loss in a kainate-induced model of TLE in rats by attenuating lipid peroxidation (Baluchnejadmojarad and Roghani, 2013). Similarly, baicalein is a flavonoid extracted from Scutellaria baicalensis, which suppresses ferroptosis by decreasing lipid peroxidation and inhibiting the expression of ALOX15. Consequently, the frequency of seizures and average seizure duration were significantly reduced in an iron chloride (FeCl3)-induced PTE mouse model (Li et al., 2019). In addition, both vitamin C and vitamin E decreased the lipid peroxidation levels; vitamin C was also found to restore GSH levels. Both vitamins were found to delay the onset of seizures and reduce the mortality rate in a rat model of epilepsy (Ayyildiz et al., 2007; dos Santos et al., 2011). Collectively, the regulation of iron, lipid peroxidation, and GSH/GPX4 levels have a remarkable impact on ferroptosis in epilepsy. Moreover, inactivation of the ferroptosis pathway has therapeutic effects in the treatment of epilepsy.

## Conclusion

Epilepsy is one of the most common neurological disorders and is characterized by spontaneous recurrent seizures. AEDs are the main treatment modality for patients with epilepsy. Although AEDs suppress seizures in two-thirds of all individuals, the long-term prognosis is not altered, and up to one-third of patients have drug-resistant epilepsy. Ferroptosis is a recently identified type of regulated cell death that is triggered by iron overload; it is characterized by unrestricted lipid peroxidation and subsequent membrane damage. Ferroptosis has been implicated in the pathogenesis of various diseases. Mounting evidence suggests that ferroptosis may play an important role in the pathogenesis of epilepsy and epileptic neuronal death. In this review, we outlined the pathogenesis of ferroptosis, the regulatory mechanisms of ferroptosis, related regulatory molecules targeting ferroptosis, and their effects on epilepsy. Current findings suggest that inhibition of ferroptosis is likely to be an effective therapeutic approach in patients with epilepsy. However, diverse modes of cell death often have similar signaling pathways and interact with each other, such as necroptosis, apoptosis, autophagy, pyroptosis and ferroptosis. Many recent studies have measured the concentration changes in ferroptosis-related regulatory molecules. However, unique markers to evaluate and distinguish ferroptotic and non-ferroptotic cell death are still lacking. In addition, previous studies have always focused on ferroptosis in hippocampal neurons. All of the different cell types should be tested in animal models of epilepsy as they may be important in the pathogenesis of epilepsy. At present, the detailed regulatory mechanism of ferroptosis in epilepsy has not yet been completely elucidated. Taken together, ferroptosis and its role in epilepsy require a systematic and in-depth investigation.

## Author Contributions

YC and ZY contributed to conception and design of the study. YC wrote the first draft of the manuscript. Both authors contributed to manuscript revision, read, and approved the submitted version.

## Conflict of Interest

The authors declare that the research was conducted in the absence of any commercial or financial relationships that could be construed as a potential conflict of interest.
